# Powdery Mildew Resistance Phenotypes of Wheat Gene Bank Accessions

**DOI:** 10.3390/biology10090846

**Published:** 2021-08-30

**Authors:** Antonín Dreiseitl

**Affiliations:** Department of Integrated Plant Protection, Agrotest Fyto Ltd., Havlíčkova 2787, CZ-767 01 Kroměříž, Czech Republic; dreiseitl@vukrom.cz; Tel.: +420-573-317-139

**Keywords:** *Blumeria graminis* f. sp. *hordei*, *Blumeria graminis* f. sp. *tritici*, infection response arrays, resistance postulation, single ear progenies

## Abstract

**Simple Summary:**

Bread wheat is one of the most important sources of human and animal food and powdery mildew is a serious disease of this crop. Breeding and growing resistant cultivars are an effective and environmentally friendly way of reducing the adverse impact of the disease on grain yield and quality. The main aim of this study was to detect major resistances against powdery mildew in a set of wheat accessions from the Czech gene bank and to group them according to their responses. Ear progenies of 448 varieties originating from 33 countries were inoculated with three isolates of the pathogen. One hundred and ten varieties showed resistance to at least one isolate and 59 varieties were resistant to all three isolates. Resistance to the three isolates was present mostly in varieties of Northwest Europe and was more than three times more frequent in spring than in winter wheats. Results will facilitate a rational and practical approach of breeding new wheat cultivars using this set of gene bank accessions as recipients of novel genes from wheat-related species and accumulate minor resistance genes to improve resistance durability.

**Abstract:**

Powdery mildew (*Blumeria graminis* f. sp. *tritici*) is a common pathogen of bread wheat (*Triticum aestivum* L.), and genetic resistance is an effective and environmentally friendly method to reduce its adverse impact. The introgression of novel genes from wheat progenitors and related species can increase the diversity of disease resistance and accumulation of minor genes to improve the crop’s resistance durability. To accomplish these two actions, host genotypes without major resistances should be preferably used. Therefore, the main aim of this study was to carry out seedling tests to detect such resistances in a set of wheat accessions from the Czech gene bank and to group the cultivars according to their phenotype. Ear progenies of 448 selected cultivars originating from 33 countries were inoculated with three isolates of the pathogen. Twenty-eight cultivars were heterogeneous, and 110 cultivars showed resistance to at least one isolate. Fifty-nine cultivars, mostly from Northwest Europe, were resistant to all three isolates were more than three times more frequently recorded in spring than in winter cultivars. Results will facilitate a rational and practical approach preferably using the set of cultivars without major resistances for both mentioned methods of breeding wheat cultivars resistant to powdery mildew.

## 1. Introduction

Plant diseases cause substantial losses in crop production and compromise food safety due to the presence of pesticides and toxins [1]. Bread wheat (*Triticum aestivum* L.) is one of the most important sources of human and animal food. Powdery mildew, caused by the biotrophic airborne fungal pathogen *Blumeria graminis* f. sp. *tritici* (*Bgt*) is a serious disease of wheat in most parts of the world that reduces yield and quality [2]. Breeding and growing resistant cultivars are an effective and environmentally friendly way of reducing the adverse impact of mildew. However, as is the case with barley mildew (*Blumeria graminis* f. sp. *hordei* = *Bgh*) [3], the use of race-specific resistance in wheat is not durable because evolutionary forces operating on cereal mildews result in extremely high diversity and adaptability of their populations [4,5]. The transfer of resistances derived from wild relatives of bread wheat could be a more effective method of disease management. Over the last decades, technologies connected with breeding have made significant strides and the knowledge gained is accelerating the identification of key resistance traits that can be efficiently transferred and applied to crop breeding programs [6].

Bread wheat is a hexaploid species that has evolved in the last 0.3–0.5 million years by spontaneous hybridization of originally diploid species and consists of three subgenomes designated as A, B, and D. The A subgenome was contributed by wild einkorn wheat *T. urartu*, the B subgenome by an unknown species closely related to *Aegilops speltoides*, and the D subgenome originated from *A. tauschii* [7]. Wheat is thus related to a range of species belonging to its primary, secondary and tertiary gene pools [8]. Introgression of novel genes from wheat progenitors and related species can increase the diversity of agronomically important traits such as disease resistance, which are invaluable in the breeding of the crop.

Research presented in this contribution is a prerequisite for a project of wheat genome-wide association study (GWAS) involved with a large-scale analysis of correlations between phenotypes of many accessions and aimed to identify genes associated with drought and frost tolerance, resistance to ear fusariosis and genes affecting developmental stages of plants and especially flowering time.

The goal of this study was to define resistance of potential genotypes of wheat, i) as recipients for introgressing powdery mildew resistance derived from *Triticum militinae* [9] and *T. monococcum* [10], and ii) for accumulating minor resistance genes from the tested cultivars. The aim of the current tests was to detect resistances based on major genes to powdery mildew at the seedling stage and to group the cultivars according to resistance phenotype.

## 2. Materials and Methods

The following methods, especially in Section 2.2 and Section 2.3, are similar to those previously described [11].

### 2.1. Plant Material and Pathogen Isolates

Ear progenies of 448 cultivars selected from the Czech wheat gene bank and multiplied in rows in the field were studied. For seedling resistance tests two domestic (Czech) and one Russian isolate of *Bgt* were used. Isolate E originated from a wheat field between Dačice and Chlumec in May 2018, isolate Tm-258 was collected from an experimental line Tm-258 in Olomouc in May 2011, and isolate Galina was recovered from a cultivar of the same name in the St. Petersburg area in July 2018. Their virulence/avirulence patterns were determined on eight selected wheat cultivars (see later). The isolates were multiplied on leaf segments of susceptible winter wheat AF Jumiko and fresh conidia were used for inoculation.

### 2.2. Testing Procedure

For in vitro resistance tests about five seeds of each accession were sown in pots (80 mm diameter) containing a gardening peat substrate and placed in a mildew-proof greenhouse under natural daylight. The primary leaves were excised when the second leaves were emerging, and leaf segments 20 mm long were cut from the middle part of healthy fully expanded leaves. Five segments of each accession were deposited on the surface of media (0.8% water agar containing 40 mg^−L^ of benzimidazole—a leaf senescence inhibitor) in a 150 mm Petri dish. Leaf segments were placed next to each other with their adaxial surfaces facing upward.

For inoculation, a cylindrical metal settling tower of 150 mm diameter and 415 mm in height was used and a dish with leaf segments was put at the bottom of the tower. Conidia of each isolate, taken from leaf segments of the susceptible cultivar with fully developed pathogen colonies, were shaken onto a square piece (40 × 40 mm) of black paper to visually control the amount of inoculum deposited. Then, the paper was rolled to form a blowpipe, and conidia of the isolate were blown through a side hole of 13 mm diameter in the upper part of the settling tower over the Petri dish at a concentration of ca. 20 conidia mm^−2^. Before inoculation with another isolate the settling tower and other tools were sterilised with ethyl-alcohol 96%. The dishes with inoculated leaf segments were incubated at 18 ± 1 °C under artificial light (cool-white fluorescent lamps providing 12 h light at 30 ± 5 μmol m^−2^ s^−1^).

### 2.3. Evaluation

**Figure 1:**
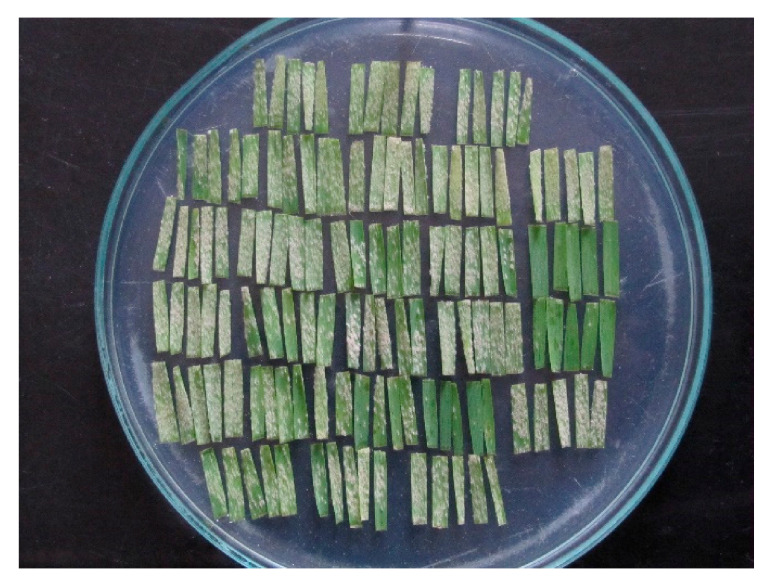
Twenty-six wheat cultivars each represented with a pentad of leaf segments seven days after inoculation with a *Blumeria graminis* f. sp. *tritici* isolate.

Seven days after inoculation, infection response (IR = phenotype of accession x isolate interaction) on the adaxial side of leaf segments (Figure 1) were scored on a scale 0–4, where 0 = no mycelium and sporulation, and 4 = strong mycelial growth and sporulation [12]. IRs 3, 3–4 and 4 were considered susceptible. Each cultivar was tested once with the three isolates and subsequently with a colony isolate derived from each of the three isolates. If there were significant differences in IRs between them, additional tests were done.

### 2.4. Numerical Designation of Resistance Groups

Resistance phenotypes characterised by IRs of a cultivar to the three *Bgh* isolates formed an infection response array (IRA) used for numerical designation of its resistance group. If there was a resistant response to a corresponding isolate, the first isolate was given the value 1 (2^0^), the second isolate = 2 (2^1^), and the third isolate 4 (2^2^). Therefore, a digit can have a value from 0 (no resistance to any of the three isolates) up to 7 (= 1 + 2 + 4) denoting resistance to each of the three isolates. The resulting number (reverse-octal) defines phenotypic classification of the resistance/susceptibility pattern of each cultivar and its resistance group.

## 3. Results

Four hundred and forty-eight wheat cultivars were tested, of which 422 were winter and 26 spring growth habit; 420 cultivars were homogeneous whereas 28 showed heterogeneous IRAs when two or more IRs were detected in one or more cultivar-isolate interactions (Table 1).

Major resistance to powdery mildew was found in 110 homogeneous accessions, 95 of which were resistant to the Tm-258 isolate, 71 resistant to the isolate E and 92 cultivars to the Galina isolate. According to their responses to the isolates, homogeneous cultivars were divided into eight groups (Table 2). Twenty-one cultivars were resistant to one isolate (sum of groups 1, 2 and 4), 30 were resistant to two isolates (groups 3, 5 and 6) and there was resistance to all three isolates in 59 cultivars (group 7). Susceptibility to all isolates was the most frequent, detected in 310 cultivars (group 0).

Cultivars Arktis, Novosibirskaya 2 and Ronsard (Table 2) were susceptible only to isolate Tm-258 (resistance group 6), Gourmet and the remaining 24 cultivars were susceptible solely to isolate E (resistance group 5) and Magnifik and MV Zelma were susceptible only to isolate Galina (resistance group 3). These three groups of cultivars can characterise the virulence of the three isolates used.

Cultivars originated from 33 countries, including two from ‘pre-1989′ countries (Czechoslovakia—six cultivars and the German Democratic Republic—one cultivar, Hadmerslebener Qualitas). European cultivars predominated (410), while there were only 16 cultivars from non-European countries (Argentina, Canada, Japan, Korea, Kazakhstan, Kyrgyzstan and the USA). Cultivars originating from Russia (19) and Turkey (1) were not assigned to either group and the origin of two cultivars (Alomar and Gourmet) is unknown. The most frequent cultivars were from Germany (112), the Czech Republic (104) and France (70).

Cultivars resistant to all three isolates were found in 10 national groups (Table 3), most commonly from the Netherlands (37.5%), Denmark (33.3%), Germany (25.5%), France (21.5%), Sweden (20.0%) and Great Britain (12.5%). Such resistance was also found in nine cultivars from three Central European countries (an average of 7.0%). One (SU—Kae no. 169) out of two cultivars from Korea, and one (Alomar) of two cultivars of unknown origin were also resistant to the three isolates. No other cultivar from the remaining 23 countries showed such resistance. Resistance to all three isolates was found in 48 out of 363 (13.2%) homogeneous European winter cultivars compared with 9 out of 22 (40.9%) European spring cultivars (both groups differ significantly at α = 0.01 for binomial distribution).

## 4. Discussion

Pathogen resistance can be identified with genetic analyses based on Mendel’s laws of inheritance and validated for plant resistance to causal agents of diseases [13], based on a gene-for-gene model [14] using sets of selected pathotypes (resistance gene postulation), or with combinations both these methods [15]. The precondition for the first two methods is genotypic purity of the accession or population after crossing.

Octal notation has been developed [16] and recommended [17] for designating pathotypes (races) of plant pathogens since it clearly concentrates information about their virulence/avirulence patterns. For the same reason it was later adopted for denoting host resistance/susceptibility responses [18] and is now also used here.

Gene bank accessions are commonly characterized by high genotypic heterogeneity [11]. Therefore, ear progenies of the tested cultivars were grown for this project. Despite these precautions 28 (6.2%) heterogeneous accessions were revealed possibly resulting from outcrossing during multiplication or mechanical admixtures with other genotypes or during the preparation of accessions. These heterogeneous cultivars could not be assigned to any of the eight resistance groups. A more recent method for identifying resistance in heterogeneous hosts has been developed [15] particularly fit for cereals, which combines genetic analysis and postulation of resistance genes by clusters of selected pathogen isolates. However, this method was not used in the present investigation.

Host-pathogen relationships are binary (resistant or susceptible) and by using three isolates a maximum of eight (2^3^) resistance groups could theoretically be identified [16]. The results confirmed the diversity and suitability of the selected *Bgt* isolates because the cultivars could be separated into all eight groups that would theoretically be expected from their phenotypes (resistance responses). This is not surprising because in the Central European population of the “sister” pathogen *Bgh* the highest biological diversity among all known plant pathogens was found when 226 isolates belonged to 224 pathotypes [5]. With reference to the GWAS project, cultivars from group 0 (no major resistance detected) will be used as preferred recipients of resistances derived from the relatives of bread wheat and also in the search for resistance based on minor genes.

The set of European homogeneous cultivars consisted of two diverse subsets—363 winter and 22 spring wheats. Nevertheless, the proportion of spring wheat resistant to all three isolates (40.9%) was more than three times higher than winter wheats (13.2%) suggesting that mildew resistance has been a higher priority in breeding spring compared with winter wheat. This is possibly because spring wheat is most vulnerable when its emergence in the field coincides with a high pathogen inoculum spreading from established fields of neighbouring non-resistant winter wheats. This influx of inoculum leads to higher yield losses of spring cultivars and emphasises the importance of resistance breeding programmes.

The analysis of cultivar origin characterized by resistance to all three isolates showed that such resistance was present in 48 out of 203 cultivars of Northwest Europe but in only 9 out of 182 other European wheats (significant difference at α = 0.01 for binomial distribution). This region has more suitable conditions for pathogen development such as high humidity, milder winters with continuing crop growth and more temperate summers, all of which are conducive for pathogen reproduction and crop damage. To protect wheat from powdery mildew by breeding resistant cultivars must be a priority in the maritime climate of Northwest Europe compared with other regions [19].

Our results show that European bread wheats and mainly those originating from Northwest Europe are rich in major resistances. For example, from 146 Chinese commercial wheat cultivars and breeding lines tested with one *Bgt* isolate only 15.1% were resistant, whereas here 23.6% older accessions were resistant to three isolates. However, 16.4% of those genotypes showed resistance at the adult-plant stage [20]. These results indicate that other, possibly minor non-specific genes are present in the wheat germplasm.

The public demand for reducing chemical applications and especially those for food production, emphasizes the need for limiting the effects of crop diseases using genetic resistance. According to the adaptability of pathogens [4,21], plant resistance can be divided into two groups [22]. The first group is represented by major genes, which are highly efficient in the absence of virulent pathotypes, but the resistance of most major genes is rapidly overcome by the evolution within the pathogen population [23]. Such resistance, including wheat-powdery mildew pathosystem, is intensively studied and a better understanding could lead to its more widespread use in the breeding of cultivars [24,25]. Another promising way to use major genes is to obtain durable resistance based on loss-of-function *mlo* gene [26] widely used against barley powdery mildew [27]. Nevertheless, it is unclear how this recessive gene can express the resistance in hexaploid wheat.

The second group of resistances, which includes minor genes, is characterized by lower efficacy because it allows limited reproduction of the pathogen [28,29], but is usually more resistant to pathogen adaptation [30]. Kang et al. [31] summarized details of many resistance genes of both groups, including introgressions from about 30 species of *Triticum* and other relatives and demonstrates the potential of diverse wheat resistance resources to powdery mildew. At the same time, there are plenty of well-characterised low-effect genes in *T. aestivum* itself [32,33]. These were sufficiently effective in the United Kingdom even when winter wheat had been intensively cultivated under conditions favourable for powdery mildew infection [34] and is appropriate for reducing powdery mildew infection in the field [35]. The results presented in this contribution provide a sound basis for increasing powdery mildew resistance in wheat breeding using the tested cultivars as recipients of novel genes from wheat-related species and/or as a means to accumulate minor resistance genes for improving resistance durability.

## 5. Conclusions

-  Breeding and growing resistant cultivars are an environmentally safe and cheap way of disease management.
-  In 448 older cultivars from the Czech wheat gene bank, resistance phenotypes against powdery mildew were studied. Despite testing ear progenies 28 accessions were heterogeneous because they were composed of different genotypes.
-  In total, 110 cultivars were resistant to one or more of the three isolates that were used, and they could be separated into eight resistance groups.
-  Fifty-nine cultivars mostly from Northwest Europe were resistant to all three isolates. The frequency of such cultivars was more than three times higher in spring than in winter wheat accessions. This indicates that more favourable conditions for pathogen development occur in maritime regions and breeding spring wheat with mildew resistance is a priority in these environments.
-  In winter wheat, the use of well-characterized low-effect resistance genes (minor genes) against powdery mildew is sufficiently effective.
-  The potential of 30 *Triticum* species and near relatives as valuable resistance sources can be considered.

## Figures and Tables

**Table 1:** Four hundred and forty-eight wheat cultivars, their growth type, country of origin and response to three powdery mildew isolates coded in reverse-octal notation (resistance group).

Cultivar ^1^	O ^2^	G ^3^	Cultivar	O	G	Cultivar	O	G
Activus	2	5	Farabi	20	0	Norstar	6	0
Addict	13	7	Faunus	2	5	Novosibirskaya 2	27	6
AF Jumiko	8	0	Faustus	10	7	Novosibirskaya 3	27	0
Airbus	13	0	Federer	8	0	Novosibirskaya 32	27	0
Akasabishirazu 1	19	0	Fenomen	13	7	Novosibirskaya 40	27	0
Akteur	10	0	Feria	13	0	Odesskaja 16	32	0
Aladin	10	0	Fermi	13	7	Odesskaja 66	32	0
Alana	8	0	Filon	13	4	*Odeta*	8	7
Albertus	2	7	Fisht	27	0	Oska	8	0
Alceste	24	7	Florett	13	7	Pajbjerg 184	11	0
Alexander	10	0	Florian	10	0	Pankratz	10	0
Alibaba	10	0	Forhand	8	0	Pannonia NS	28	0
*Alicia*	8	5	Franz	10	7	Papageno	2	7
Aliya	20	0	Frisky	13	4	Partner	13	7
Alka	8	0	Gallio	2	5	Patras	10	0
Alomar	34	7	Gaudio	10	5	Penalta	8	0
Alpine Neuzucht	2	0	Genius	10	0	Penelope	8	0
Altigo	13	h	Globus	10	0	Petrus	10	0
Amandus	2	0	Gordian	17	5	*Pexeso*	8	1
Anara	20	h	Gourmet	34	5	Pilgrim PZO	10	7
Andrejka	8	0	Grafton	14	0	Pionier	10	0
Angelus	2	0	Graindor	13	0	Pitbull	10	7
Aniya	20	h	Grana	25	0	Plantahof 3	17	0
Annie	8	0	*Granny*	8	5	Ponticus	10	7
Antonius	2	0	Grizzly	8	0	Porthus	10	7
Apache	13	0	Hadmerslebener Q.	9	0	Postoloprtska Pres.	7	0
Apanage	13	4	Hana	8	0	Potenzial	10	0
Apertus	10	0	Hanacka Osinata	8	0	Praskoviy	27	0
Apollo	24	0	Hanswin	17	0	Preciosa	24	0
Apostel	10	7	Hedvika	24	0	Premio	13	0
Arina	17	0	Henrik	13	0	Prestizh	27	h
Arkadia	25	0	Hermann	13	0	Prince Leopold	3	1
Arkeos	13	0	Hewitt	24	7	Princeps	10	0
Arktis	10	6	Hondia	25	0	Proteus	13	0
Artist	10	0	Chevalier	10	0	Pyselka	8	0
Asta	8	0	Chevignon	13	7	*Quintus*	8	5
Astella	7	0	Chiron	10	h	Raduza	8	0
Astet	32	0	Chlumecka 12	8	0	Rapsodia	14	0
*Astrid*	8	5	Ibarra	8	0	Rassad	20	0
Athlon	13	7	Illusion	8	h	Rebell	10	0
Atlas 66	33	0	Immendorfer Kolben	2	0	Regina	8	1
Atomic	10	0	Inspiration	10	0	*Registana*	8	5
Attraktion	10	7	Iron	30	0	Renan	13	0
Atuan	10	7	IS Agape	29	0	Rexia	8	0
Avenue	13	5	IS Conditor	29	0	RGT Cesario	13	0
Axioma	10	0	IS Danubius	29	0	RGT Matahari	8	0
Bagou	13	7	IS Escoria	29	0	RGT Mobidick	8	0
Bakfis	8	0	IS Gordius	29	0	RGT Premiant	8	0
Balan de Figanesti	26	0	*IS Jarissa*	29	7	RGT Reform	10	h
Baletka	8	0	IS Laudis	29	1	RGT Sacramento	13	0
Balitus	2	5	Ivanovskaja 12	32	0	Rheia	8	0
Bamberka	25	0	Izalco CS	13	0	Rivero	10	7
Banderola	25	0	*Izzy*	8	7	Rockefeller	10	7
Bankuta	16	0	Jensen	11	0	Rodnik Tarasovskij	27	0
Bankuti 8000	16	0	Jindra	8	2	Ronsard	13	6
Banquet	8	0	Johnson	13	7	Rosatch	17	0
Barabas Fele	16	0	Jubile II	3	0	Rumor	10	0
Baracuda	10	7	Judita	8	0	Rumunka	5	0
Barbarossa Podol	25	0	Julie	8	0	Rusalka	4	0
Bardan	17	0	Julius	10	0	Rytmus	8	0
Bardotka	8	0	Juna	8	h	Safari	10	0
Barroko	14	h	*Kabot*	10	5	Sailor	10	7
Barryton	13	0	Kanhard Sel. Buck	1	0	Sakura	8	0
Basilio	13	0	Kanzler	10	0	Sally	8	0
Batis	10	h	Kasticka Osinatka	8	0	Samanta	8	0
Batkan Krasnaya	21	0	Kelvin	24	h	Samara	8	0
BC Anica	15	h	*Kitri*	8	7	Samurai	10	0
BC Darija	15	5	Kodex	10	0	San Pastore	18	0
BC Lira	15	0	Kometus	10	5	Sarka	8	0
Beduin	13	0	Kompass	10	0	Sarmund	10	0
Bekend	8	7	Korneuburger	2	0	Saskia	8	0
Belgrade 1	28	0	Korneuburger Gran.	2	0	Saxo	30	0
Benschmark	11	7	Kosutka	8	0	*Seance*	8	5
Bermude	13	0	Kredo	10	7	Secese	8	0
Bernstein	10	1	Kulundinka	27	0	Seladon	8	0
Bezostaja 1	27	0	Kurt	10	7	Sepia	13	4
Bienfait	13	0	KWS Dacanto	10	0	Sepstra	10	0
Biscay	10	0	KWS Emil	10	5	*Septima*	8	5
Bizel	13	0	KWS Eternity	10	0	Seu Seun 8	22	0
Bodycek	8	0	KWS Ferrum	10	0	Sheriff	11	h
Bohemia	8	0	KWS Fontas	10	0	Sida	8	0
Boisseau	13	7	KWS Loft	10	7	Sila	27	0
Bonanza	10	7	KWS Magic	10	0	Silvanus	29	0
Boregar	13	0	*KWS Mairra*	10	7	Simila	8	2
Botagoz	20	0	KWS Montana	10	0	Siria	7	0
Brea	8	0	KWS Ronin	10	0	Skorpion	8	0
Brentano	10	h	KWS Santiago	14	7	Slovenska 777	8	0
Brigala	27	0	KWS Silverstone	14	0	Smaragd	10	0
Brilliant	10	0	KWS Smart	10	0	Sofolk	13	2
Brokat	10	0	Landsknecht	10	0	Sofru	13	0
Buteo	10	0	Laurier	13	h	Solindo	13	7
Butterfly	8	0	Lavantus	10	0	Somtuoso CS	13	0
Calisol	13	0	Lavoiser	13	h	Sonergy	13	0
Calumet	13	0	Lear	14	0	Sosthene	13	0
Caphorn	14	1	Legenda Mironovsk.	32	h	Sparta	8	0
Capone	10	0	*Leguan*	8	5	Spontan	10	h
Carmina	8	0	Lemaire 4	13	0	Stadium	13	7
Cecilius	2	0	Lena	8	h	Steffi	8	h
Cellule	13	0	*Lennox*	10	5	Stupicka Bastard	8	0
Ceska Presivka	7	0	LG Imposanto	10	0	SU Kae no. 169	22	7
Ceylon	12	0	LG Magirus	10	0	Sulamit	8	2
Cimrmanova Rana	8	0	LG Mocca	10	7	Sultan	8	0
Citrus	10	0	*Libertina*	8	5	*Sumai 3*	19	0
Clever	14	0	Litera	26	0	Svitava	8	0
Cocoon	13	0	Lithium	13	h	*SW Kadrilij*	30	7
Collector	13	0	Loosedorfer Winter.	2	0	SY Alteo	13	0
Colonia	10	7	Lorien	8	0	SY Mattis	13	0
Complet	10	0	*Lotte*	8	h	SY Passport	10	7
Complice	13	0	Lovaszpatonai 157	16	0	Tabasco	10	7
Conexion	13	0	Lovrin 13	26	0	Tarasovskaya Ostist.	27	0
Corsaire	13	0	Ludwig	2	0	Tau	27	0
Coutiches	13	0	Lukullus	2	5	*Tercie*	8	5
Cubus	10	1	Luna	25	0	Terroir	13	0
*Dafne*	8	7	Magister	10	0	Tervel	4	0
Dagmar	8	0	Magnifik	12	3	Tiguan	13	0
Dalmatia 2	15	0	Magno	17	0	Tilman	13	7
Dancing Queen	8	7	Maira	20	0	Timing	13	4
Dankowska Biala	25	0	Manitou	10	7	Tir	31	0
Darwin	10	0	Mara	8	0	Tobak	10	0
Diadem	8	0	Marquardt II	10	0	Todireshti	23	0
Dichter	10	0	Master’s New Y.	14	0	Tonnage	11	7
Dmitriy	27	0	Matchball	8	0	Torp	11	5
Drifter	10	0	Matylda	8	0	Tosca	8	0
Dromos	10	0	Meritto	8	0	Tower	24	0
Duecentodieci	18	0	Mescal	10	0	*Trappe*	10	0
Dulina	8	0	Messi	8	0	Trumf (Heines IV)	10	0
Ebi	10	0	Midas	2	0	Tuerkis	10	0
Edgar	10	0	Minhardi	33	0	Tulecka	25	0
Elan	10	0	Miranda	26	0	Turandot	8	0
Elixer	13	h	Mironovska	8	h	Tvorec	27	0
Elly	8	0	Mladka	8	0	*Tybalt*	10	7
Emilio	2	0	Mona	8	0	Uljanovka	27	2
Energo	2	0	Montaldo	17	0	Urup	27	h
Epi d_Or	13	7	Mozes	10	1	Valticka Osinata B	7	0
*Epos*	10	7	Mulan	10	0	*Vanek*	8	h
Eroica	30	0	Mutic	13	0	Vanessa	8	0
Ershovskaya 10	27	0	MV Beres	16	0	Venistar	29	0
Estevan	2	0	MV Bodri	16	0	Viki	8	0
Estica	24	0	MV Kolompos	16	0	Viriato	13	2
Estivus	10	0	MV Nador	16	0	Vlasta	8	0
Etana	10	0	MV Nemere	16	h	Volodarka	32	0
Etela	8	0	MV Pengo	16	0	Vouska z Tremos.	8	0
Etuos	10	7	MV Zelma	16	3	Weibulls Trond	30	0
Euclide	13	0	Nakskov	11	0	WPB Calgary	24	7
Eurofit	2	0	Nelson	10	h	Zdar	8	1
Event	10	0	Nikol	8	0	Zeppelin	10	0
Evina	13	0	Nordika	8	0	Zidlochovicka Osin.	7	2
Fabius	2	0	Nordkap	10	7	Zora	8	0
Fairway	13	0	Norin	10	0	-	-	-
Fakir	10	0	Norin 40	19	0	-	-	-

^1^ Spring wheats are written in italics. ^2^ Country of origin: 1 ARG Argentina, 2 AUT Austria, 3 BEL Belgium, 4 BGR Bulgaria, 5 BIH Bosnia and Herzegovina, 6 CAN Canada, 7 CSK Czechoslovakia, 8 CZE Czech Republic, 9 DDR German Democratic Republic, 10 DEU Germany, 11 DNK Denmark, 12 FIN Finland, 13 FRA France, 14 GBR Great Britain, 15 HRV Croatia, 16 HUN Hungary, 17 CHE Switzerland, 18 ITA Italy, 19 JPN Japan, 20 KAZ Kazakhstan, 21 KGZ Kyrgyzstan, 22 KOR Korea, 23 MDA Moldavia, 24 NLD Netherlands, 25 POL Poland, 26 ROM Romania, 27 RUS Russia, 28 SRB Serbia, 29 SVK Slovakia, 30 SWE Sweden, 31 TUR Turkey, 32 UKR Ukraine, 33 USA United States of America, 34 Unknown. ^3^ Resistance group (0–7), h = heterogeneous.

**Table 2:** Infection response arrays (IRAs) of 420 homogeneous wheats represented by eight model cultivars separately inoculated with three isolates of powdery mildew and octal notation of infection responses to determine their resistance group.

Model Wheat Cultivar	Powdery Mildew Isolates			Octal Notation (Group)	Group Frequency (*n*)
	Tm-258 (2^0^ = 1)	E (2^1^ = 2)	Galina (2^2^ = 4)		
AF Jumiko	s	s	s	0	310
Pexeso	r	s	s	1	9
Sulamit	s	r	s	2	7
Magnifik	r	r	s	3	2
Apanage	s	s	r	4	5
Gourmet	r	s	r	5	25
Ronsard	s	r	r	6	3
Dancing Queen	r	r	r	7	59

r = resistant, s = susceptible.

**Table 3:** The number of wheat cultivars according to their country of origin and designation as heterogeneous, susceptible or resistant to three powdery mildew isolates.

Country of Origin ^1^	Number of Cultivars					Resistant (%) ^2^ (Group 7)
	Total	Heterogeneous	Susceptible (Group 0)	Resistant (Group 7)	In groups 1–6	
NLD	9	1	5	3	0	37.5
DNK	7	1	3	2	1	33.3
DEU	112	6	70	27	9	25.5
FRA	70	5	42	14	9	21.5
SWE	5	0	4	1	0	20.0
GBR	9	1	6	1	1	12.5
SVK	9	0	7	1	1	11.1
AUT	23	0	16	2	5	8.7
CZE	104	7	75	6	16	6.2
KOR	2	0	1	1	0	50.0
Unknown	2	0	0	1	1	50.0
ARG	1	0	1	0	0	0
BEL	2	0	1	0	1	0
BGR	2	0	2	0	0	0
BIH	1	0	1	0	0	0
CAN	1	0	1	0	0	0
CSK	6	0	5	0	1	0
DDR	1	0	1	0	0	0
FIN	2	0	1	0	1	0
HRV	4	1	2	0	1	0
HUN	11	1	9	0	1	0
CHE	8	0	7	0	1	0
ITA	2	0	2	0	0	0
JPN	3	0	3	0	0	0
KAZ	7	2	5	0	0	0
KGZ	1	0	1	0	0	0
MDA	1	0	1	0	0	0
POL	9	0	9	0	0	0
ROM	4	0	4	0	0	0
RUS	19	2	15	0	2	0
SRB	2	0	2	0	0	0
TUR	1	0	1	0	0	0
UKR	6	1	5	0	0	0
USA	2	0	2	0	0	0

^1^ Full names of the countries can be seen in a footnote to Table 1. ^2^ Heterogeneous cultivars were not included.

## Data Availability

All relevant data are presented in this contribution.
